# The Elderly Population in Iran: An Ever Growing Concern in the Health System

**Published:** 2012

**Authors:** Maryam Noroozian

**Affiliations:** 1 Memory & Behavioral Neurology Department, Roozbeh Hospital, Tehran University of Medical Sciences (TUMS), Iran.

**Keywords:** Old Population, Iran, Dementia, Alzheimer's Disease

## Abstract

The population ages 65 and over is expected to grow very rapidly in all parts of the world. Over the next decades, the elderly population is projected to grow much more quickly than the total population in all parts of the world.

At the global level, the number of those over age 60 is projected by the UN Population Division to increase from just under 800 million in 2011 (representing 11% of world population) to just over 2 billion in 2050 (representing 22% of world population). World population is projected to increase 3.7 times from 1950 to 2050, but the number of those aged 60 and over will increase by a factor of nearly 10. Among the elderly, the “oldest old” – i.e., those aged 80 and over – is projected increase by a factor of 26.

Accompanying these projected increases in elder shares throughout the world is another salient trend: the “compression of morbidity”. Anti-aging technologies – from memory-enhancing drugs to high-tech joint replacements – and healthier lifestyles have not merely increased longevity but have also made old age healthier.

Although population aging is occurring in both developed and developing countries, the most rapid aging is taking place primarily in relatively newly industrialized or developing countries.

Population aging generates many challenges and sparks concerns about the pace of future economic growth, the operation and financial integrity of health care and pension systems, and the well-being of the elderly.

The key is adaptation on all levels: individual, organizational, and societal.

***“Older people are an enormous asset to our societies, not only because a large majority of them continue to work in old age, but also because they convey social values to younger generations”***

***Dr. Wesum***

***Asia/Pacific Regional Conference on Ageing 2012***

## Definition of an older or elderly person

According to WHO definition, most developed world countries have accepted the chronological age of “65” years as a definition of 'elderly' or older person but there are many studies which consider the age of “60” for developing countries.

## The World is Graying

The growth of the elderly population is inevitable, and will occur worldwide.

This rapid population growth will have adverse effects on socioeconomic advancement and the health of older people.Over the last 60 years, the average life expectancy, which leapt from about 48 years in the early 1950s,has been extended to about 68 in the first decade of the new century(2). 

The expansion of the number of aged individuals in the population will unavoidably be accompanied by an increasing number of age-related disorders, including Alzheimer’s disease (AD), Vascular Dementia (VD) and Mild Cognitive Impairment (MCI)(3).

**Figure 1:**
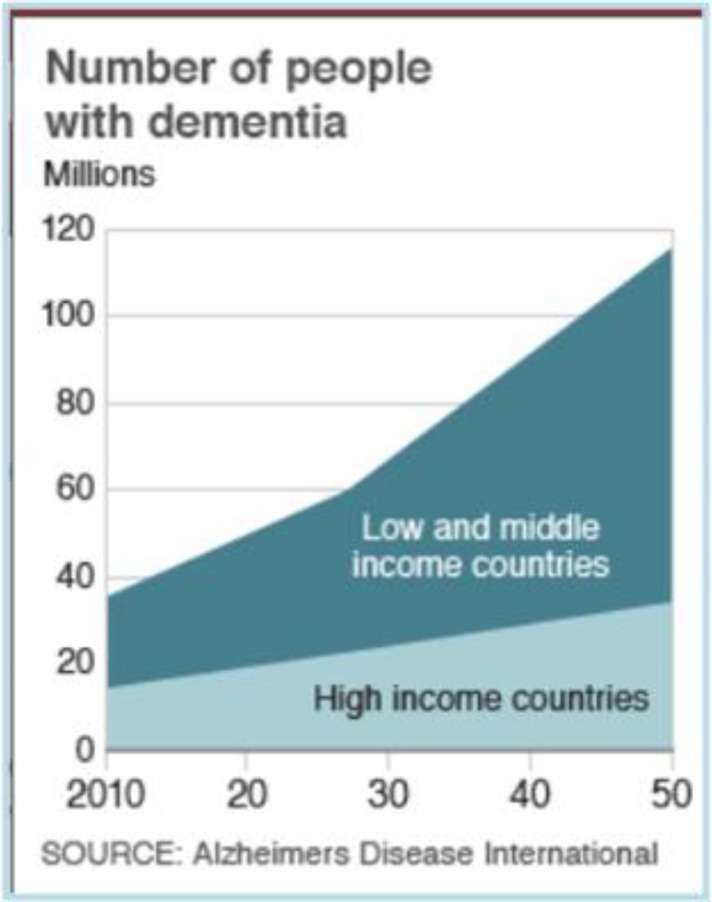
Worldwide number of people with Dementia

In the World Alzheimer Report 2009, Alzheimer Disease International (ADI) estimated that 36 million people worldwide with dementia, with numbers doubling every 20 years to 66 million by 2030, and 115 million by 2050. Much of this increase was found to be in low and middle income countries; 58% of those with dementia currently live in low and middle income countries, rising to 71% by 2050. The worldwide cost of dementia was estimated around US$604 billion in 2010(4) (Figure 1).

## Aging in Asia

All across Asia, the process of population aging is occurring much more rapidly than it did in Western countries, and it will occur in some Asian countries at a much earlier stage of economic development (Figure2).

Considering the report of Asia/Pacific Regional Conferenceon Ageing 2012, the share of older people inthe total population is growing at a faster pace, never seen anywhere inthe world (5).

By2025 there will be over 700 million people over the age of 60. This rapid increase in the percentage share of older peoplein the total population means a reduction of the percentage of the workingadults, and therefore a reduction of support base for older people and an increase in their dependency ratio.

Some of the consequences of such a demographic change in Asia are:

- **The rapid increase of the older women, and especially the older-old women:**

This is a major challenge for old age care crisis as women are the primary caregivers in the family. Older women are, in general, more flexible in their relationships and outlook and are thus easy to engage and provide care. At the same time, the older-old women constitute the larger group who need the most acute care as most older women outlive their spouses into their 80s and 90s, many of them widowed and living alone. Their vulnerability and therefore need for care is heightened by the fact that oldest-old women are also less educatedon average than males, have fewer assets in marriage than men and are more likely to be poor and without accumulated savings.

- **Internal and global migration and urbanization:**

Elderly care are been further aggravated by the migration of young adults, both women and men, in search of employment. Migration is a drain on the availability of adult caregivers therefore the older parents are being left with a double burden.

- **Changes in traditional forms of care:**

Then there are also on-going changes to family structures that see a declining size of the nuclear family,and a greater number of older family members living separately. Even though some of olderpeople who currently live alone expressed the advantage of this lifestyle inthat they spend less time on their children’s family needs, they could spendmore time on their own hobbies, and they were less likely to be stressed byadult children or by daughter-in-laws who had different approaches to child raising or other family matters, the disadvantages become apparent, though, when the older people become frail and require daily living supporter caregiving. Separate living arrangements thus create patterns of independent living, and when there is a need for caregiving, many children and grandchildren view this as an additional burden in terms of time and costs when outside caregiving support is required.

- **Mental health:**

Mental health in terms of depression and dementia are alsoa reality of ageing, and is becoming an increasing burden in the care forolder people. Considering the absence of a definite cure for AD and other degenerative causes of dementia, these devastating disorders are a major source of caregiver burden(5).

## Iran

The Islamic Republic of Iran which is located in WHO Eastern Mediterranean regionis the 18th largest country in the world in terms of area at 1,648,195 km and the 4th largest country in Asia.

According to the report of 2011 census which was the seventh national population and housing census of the country, the total population of Iran has been 75,149,669 (comprising 50.4 % male and 49.6% female) whom 71.4% were settled in urban areas (Figure2)(6).

**Figure3:**
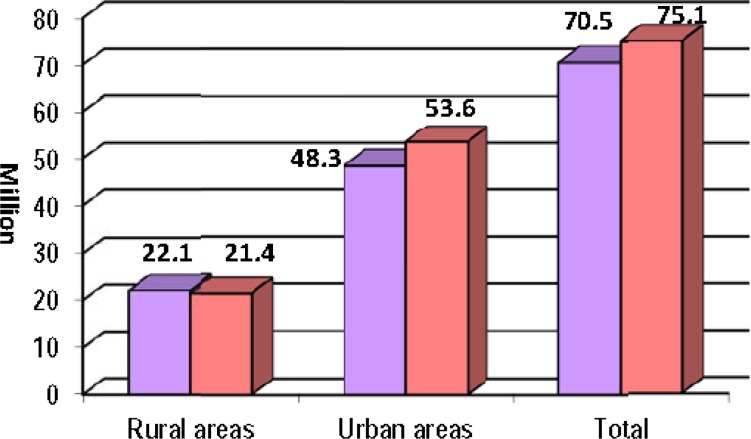
Population changes in Iran, Census 2011

In addition average household size has been decreased from 4.03 in 2006 to 3.55 in 2011. Even though this change will definitely have positive impacts on family health and economy, the negative consequence of such a change will be reflected in the caregivers’ numbers for the old parents in the future. 

The comparison between population age pyramids shows the trend of ever increasing old population in Iran during recent five years (Figure 3).

**Figure 4:**
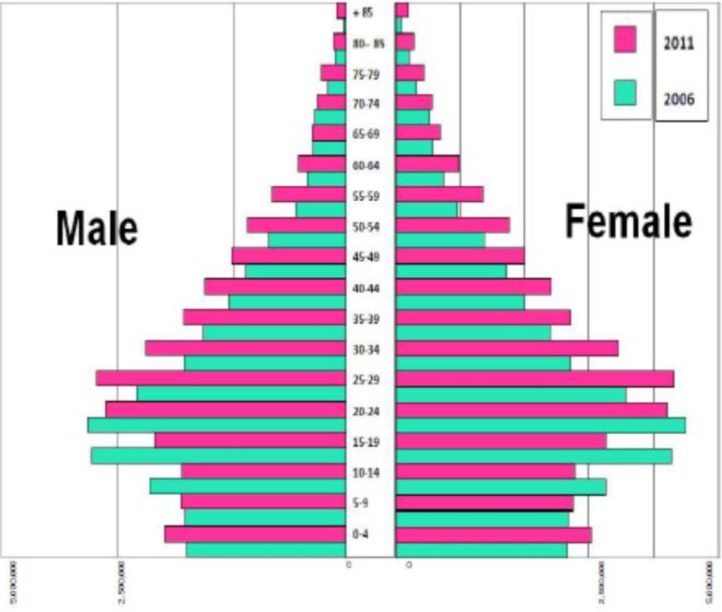
Comparison between population age pyramids in Iran from 2006 to 2011

Comparing Iran's population age pyramid in the past two decades illustrates that the structure of age pyramid is reversing. It shows that in the coming years the present young population will incline towards aging (Figure 4).

As it has been shown in Table 1, the most important demographic finding in the recent census in Iran is the remarkable change in the number of aged people which has been increased from 7.22% in 2006 to 8.20% in 2011 (Table 1).

Moreover the highest percentage is seen in the age group of 15-64 year old (70.9%) which will be followed by a huge population of old people in the next decades (Table 2).

**Table 1:** Percentage of different age groups in the elderly population in Iran during two consecutive census studies in 2006 and 2011, Iran Census 2011:

**Age group (years)**	**2006 (%)**	**2011(%)**
60 -64	2.08	2.48
65-69	1.70	1.79
70-74	1.59	1.49
75-79	0.98	1.22
80 and over	0.92	1.22
**Total percentage of old population ** **60** ** years and over**	**7.22**	**8.20**

**Table 2:** Distribution of age groups during five consecutive census studies in Iran population, 1976-2011:

**Year**	**0-14 year old (%)**	**15-64 year old (%)**	**65** ** Year old ** **andover (%)**
2011 (1390)	23.4	70.9	5.7
2006 (1385)	25.1	69.7	5.2
1996 (1375)	39.5	56.1	4.3
1986 (1365)	45.5	51.5	3.0
1976 (1355)	44.5	52.0	3.5

Increasing life expectancy is a valuable index that is considered an achievement for thehealthsysteminIranbecause of raising public awareness about age-related disorders (Table 3). 

**Table 3:** Distribution of Life Expectancy in both Gender during five consecutive Census, 1996-2011:

**Year** **Sex**	**2006(1385)**		**2011(1390)**	
	**Women**	**Men**	**Women**	**Men**
**Life Expectancy**	73.1	71.1	74.6	72.1

- **Literacy:**

One of the most significant indicators in social development is the literacy rate. Illiteracy is a crucial issue in demographic characteristics of elderly population because of its role as a known risk factor for developing AD on one side and lack of enough knowledge to prevent or control modifiable age-related disorders such as hypertension, diabetes, coronary artery disease, hypercholesterolemia, stroke, osteoporosis and depression on the other. Even though the rate of illiteracy among population with the age of 10 to 49 year has been decreased compared to the last census(Table 4), there is still no available data about the level of education in the old segment and the only findings about this index is found in the last census in 2006 (Table 5)(7).

**Table 4:** Adult (15-49 year old)literacy rate in Iran, Census 2011:

**Year**	**Percentage of literacy**
2011 (1390)	92.4
2006 (1385)	91.7

**Table 5:** Percentage of illiteracy in the population over 6 Years of age in. urban and rural regions, Iran, Census 2006:

	**Population 6-59 year old (%)**			**Population over 60 year old (%)**		
	**Male+** **Female**	**Male**	**Female**	**Male+** **Female**	**Male**	**Female**
**Total Country**	15.8	10.8	21.0	77.0	67.4	88.2
**Urban**	10.3	7.0	13.7	67.3	54.7	81.3
**Rural**	25.0	17.2	32.8	89.7	83.2	97.7

- **Dementia in Iran**

There are some important characteristics of dementia in general and AD specifically in Iran in terms of culture, public knowledge and socioeconomic aspects (7):

- **There is still not enough public knowledge about dementia and AD.**

Therefore itsearly signs may be considered as a natural consequence of aging in and patients are referred to the physicians in the moderate stage when behavioral symptoms develop.

- **The higher rate of illiteracy is a risk factor for the development ofAD** **(** 8 **)** **.**

- **Dementia still has not been a main priority in the policy making of Iranhealthsystem.**

Even though there is significant success in the detection of hypertension, diabetes, hypercholesterolemia and coronary artery disease – which are modifiable risk factors for AD and VD and there is ever increasing attention to geriatric issues, the focus of healthcare systems in many developing countries, including Iran, has been on maternal and child health services and infectious diseases in the past decades.

- **Caregivers’ burden is higher in Iran -and societies with the similar culture-as the strong emotional bonds between the family members and their elderly parents don’t let them to leave theiroldparents in the institutional homes.**

In our history and religion, taking care of the elderly has been one of the major commitments for the families, therefore,mostfamiliesare seriously opposed to let theirold patients live in institutional homes. However, as a result of increasing urbanization, large-scale migration, employment of both men and women, smaller size of families, and longer life expectancy of old people in the recent decades, a major change has been occurring in the public attitude toward this issue. This phenomenon is more apparent in metropolitan cities such as Tehran. In the cities with smaller population and more traditional and religious culture, there is still a negative attitude toward this issue.

- **The limited number of qualified day care centers for the old patients and insufficient services in most institutional homes contributeto the public attitude. **

Therefore, the proportion of elderly in these centers is very small in comparison to the total aged population of the country.

## Toward a Better Future

In order to achieve a better life for the old population and their family we should set our goals in different levels including governmental supports, health system policy making, medical education and public sector with a harmonic interaction.

**1. Policy Making**

- Costs for care for older people are manageable if governments are activein developing appropriate policies and programs that share responsibility and focus on prevention, and that support and regulate a market for care, especially caregiving, and treatments and products. In this direction developing and supporting cost effective home and community-based models of care is needed.

- Available evidence suggests that governments should ‘spend to save’ – in other words, invest now to save in the future. Economic models suggest that the costs associated with an earlier dementia diagnosis are more than offset by the cost savings from the benefits of medications for AD and caregiver interventions. These benefits include delayed institutionalization and enhanced quality of life of people with dementia and their caregivers (4).

- Enhancing the financial and insurance systems is a crucial part of patients support.
- Improving the quantity and quality of social services including day care centers and institutional homes compatible with medical standards, the needs and culture of our elderly.

**2. Health System and Early Diagnosis:**

- Earlier diagnosis allows people with dementia to plan ahead while they still have the capacity to make important decisions about their future care. In addition, they and their families can receive timely practical information, advice and support. Only through receiving a diagnosis can they get access to available medication and non-pharmacological treatments that improve their cognitive deficits, behavioral symptoms and their quality of life.
- Improving the likelihood of earlier diagnosis can be enhanced through:

a) medical practice based educational programs in primary care, b) the introduction of accessible diagnostic and early stage dementia care services (for example, memory clinics), and c) promoting effective interaction between different components of the health system.

- Developing screening centers for early diagnosis of age-related disorder withhigh mortality and morbidity.

**3. Medical Education:**

- Improving geriatric training in medical education by: offering a residency in

geriatrics; considering this specialty in continuous medical education (CME) for all specialties that are eligible for visiting old patients; and a short term of training for the general physicians.

**4. Social Awareness:**

- Raising awareness of our community about aging, age related disorders andsuccessful aging to consider their physical and mental health since youth.

- Creating new roles for the old people in society to keep their independence, to lead productive and purposeful lives, and to encourage those who are still capable to remain in the work force.

- Supporting and enhancing the traditional systems of family care through the help of support groups and social workers.

- **Other important issues** which should be addressed in a national planning for elderly population are:

- Palliative care and pain management
- Engaging the private sector
- Intergenerational education
- Age-friendly environment
- Preventing and treating disabilities
- Addressing abuse and neglect

In Iran, we are just beginning to experience a big challenge related to population aging and its consequences, while we still face many problems in the other areas of our health system. Like other developing countries, this process is occurring more rapidly than in the western countries. As indicated in the Asia’s aging. 

Population report: “Aging is occurring more rapidly than economic growth.”

## Notes

**Declaration of interest: **none
